# A holistic view of muscle metabolic reprogramming through personalized metabolic modeling in newly diagnosed diabetic patients

**DOI:** 10.1371/journal.pone.0287325

**Published:** 2023-06-15

**Authors:** Maryam Khoshnejat, Ali Mohammad Banaei-Moghaddam, Ali Akbar Moosavi-Movahedi, Kaveh Kavousi

**Affiliations:** 1 Laboratory of Complex Biological Systems and Bioinformatics (CBB), Department of Bioinformatics, Institute of Biochemistry and Biophysics (IBB), University of Tehran, Tehran, Iran; 2 The UNESCO Chair on Interdisciplinary Research in Diabetes, Institute of Biochemistry and Biophysics (IBB), University of Tehran, Tehran, Iran; 3 Laboratory of Genomics and Epigenomics (LGE), Department of Biochemistry, Institute of Biochemistry and Biophysics (IBB), University of Tehran, Tehran, Iran; 4 Institute of Biochemistry and Biophysics, University of Tehran, Tehran, Iran; University of California, Davis, UNITED STATES

## Abstract

Type 2 diabetes mellitus (T2DM) is a challenging and progressive metabolic disease caused by insulin resistance. Skeletal muscle is the major insulin-sensitive tissue that plays a pivotal role in blood sugar homeostasis. Dysfunction of muscle metabolism is implicated in the disturbance of glucose homeostasis, the development of insulin resistance, and T2DM. Understanding metabolism reprogramming in newly diagnosed patients provides opportunities for early diagnosis and treatment of T2DM as a challenging disease to manage. Here, we applied a system biology approach to investigate metabolic dysregulations associated with the early stage of T2DM. We first reconstructed a human muscle-specific metabolic model. The model was applied for personalized metabolic modeling and analyses in newly diagnosed patients. We found that several pathways and metabolites, mainly implicating in amino acids and lipids metabolisms, were dysregulated. Our results indicated the significance of perturbation of pathways implicated in building membrane and extracellular matrix (ECM). Dysfunctional metabolism in these pathways possibly interrupts the signaling process and develops insulin resistance. We also applied a machine learning method to predict potential metabolite markers of insulin resistance in skeletal muscle. 13 exchange metabolites were predicted as the potential markers. The efficiency of these markers in discriminating insulin-resistant muscle was successfully validated.

## Introduction

Type 2 diabetes mellitus is a complex progressive metabolic disease with a heterogeneous etiology. The disease has a high global prevalence and is among the top ten causes of death worldwide. According to the latest international diabetes federation report, the global prevalence of diabetes in 2019 is estimated to be 463 million people. In addition, 374 million people are at greater risk of developing T2DM [1]. Therefore, this disease has become a major concern in global health.

Insulin resistance is the main pathophysiologic feature in T2DM. The key insulin-sensitive tissues include skeletal muscle, liver, and adipose tissues. Among these, skeletal muscle is responsible for the clearance of over 75% of glucose from the bloodstream. Thus this tissue plays a great role in lowering the blood glucose level [2]. Dysfunction of muscle metabolism is associated with disturbance in glucose hemostasis and implicated in obesity, insulin resistance, and T2DM [3]. Therefore, the study of the molecular mechanisms underlying insulin resistance in skeletal muscle will improve our understanding and treatments for T2DM.

In complex and multifactorial diseases such as diabetes, systems biology provides a holistic view to study dysregulated subsystems associated with the disease in a cell. Meanwhile, genome-scale metabolic modeling serves as a context for *in silico* exploration of metabolism through a systems biology approach. Since there is no one-to-one relationship between transcription and translation, the study of gene expression data alone does not provide an accurate understanding of cellular metabolism; while, metabolic network simulation gives an in-depth insight into the molecular mechanisms involved in cellular processes. Generic metabolic networks are reconstructed based on the information encoded in the genome. This information includes all possible reactions that can occur in all types of human cells. By having gene expression data from a particular cell or disease state and integrating it into the generic model, active reactions are identified and a context-specific model can be reconstructed. Some efforts have been made to study the molecular mechanisms contributing to muscle insulin resistance using genome-scale metabolic models (GEMs). Bordbar et.al. have reconstructed a multi-tissue type GEMs and integrated gene expression data of obese and type 2 obese gastric bypass patients to study metabolic activity between these states [4]. Nogiec et.al. have applied flux balance analysis to model muscle insulin resistance metabolism in fasted to fed states. In addition, they tried to identify reactions that reproduce key features of insulin resistance by perturbing the muscle metabolic network. Nevertheless, they did not apply the integration of high-throughput data to the model [5]. Varemo et.al. have reconstructed a myocyte metabolic network and have utilized it to apply a gene-set analysis. They have found reporter metabolites and the metabolic pathways affected by differentially expressed genes in T2DM [6]. Previous studies were either small scale or no comprehensive simulation-based metabolic analysis was employed. Also, there is not any study that demonstrated metabolic disturbance at the early stages of diabetes. Since T2DM is a progressive disease, identification of disorders in newly diagnosed patients can provide significant information about the disease. It may bring us closer to identifying the main causes triggering the onset of disease progression.

**Fig 1:**
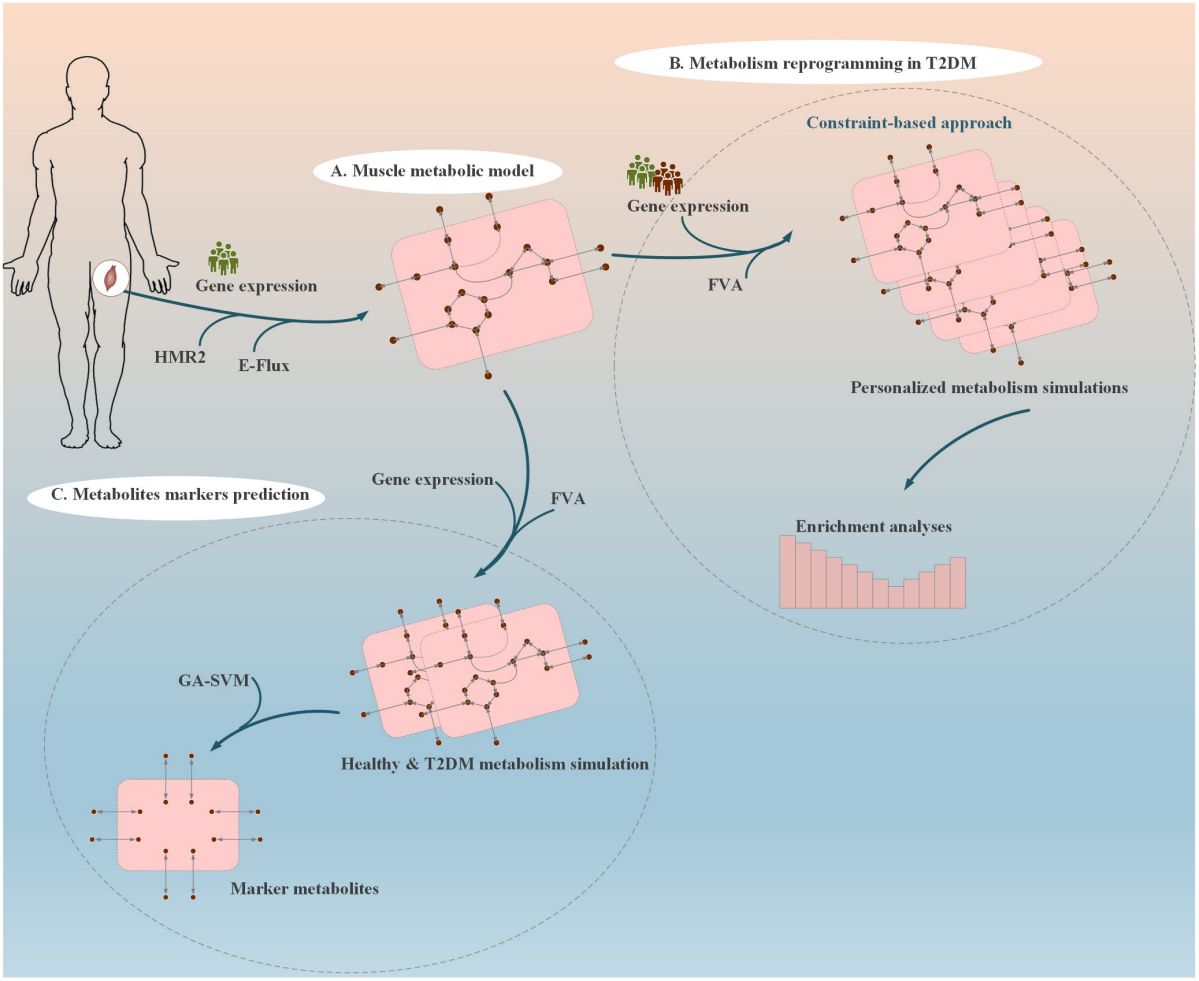
The workflow of study design. (A) The muscle-specific metabolic model was reconstructed and was employed for our following analyses: (B) Study of metabolism reprogramming in newly diagnosed T2DM patients using the personalized metabolic modeling with the constraint-based approach. (C) Prediction of potential metabolite markers of insulin resistance in muscle by applying a machine-learning technique.

In this study, the system-level metabolic analysis on the most comprehensive data from early-stage T2DM patients was performed. The samples include muscle gene expression data from newly diagnosed diabetic patients [7]. We attempted to identify early metabolic alterations associated with the disease. We first reconstructed the functional muscle-specific metabolic model. The model was employed for metabolic analyses in T2DM. We applied personalized metabolic modeling along with the constraint-based approach to study metabolic reprogramming in diabetic muscles. Moreover, potential metabolite markers related to muscle insulin resistance were predicted using a machine learning approach. Finally, we validated these markers with gene expression data from a separate study [8]. The overall workflow of this study is shown in Fig 1.

## Materials and methods

### Ethics statement

In this study, we utilized transcriptome data from the dbGaP database with the accession code phs001068.v1.p1, which was originally published in reference [7]. The authors of reference [7] obtained written informed consent from all subjects and obtained approval from the coordinating ethics committee of the Hospital District of Helsinki and Uusimaa. For validation of metabolite markers, we used muscle gene expression data of healthy and diabetic subjects from the GEO repository with the accession numbers GSE63887 and GSE81965, which were obtained from reference 8. The authors of reference [8] stated that ethical approval for the human studies, from which satellite cells were obtained, was provided by the Ethics Committee of Copenhagen and Frederiksberg Council, Denmark (KF 01-141/04). The human volunteers were given oral and written information about the experimental procedures, and all subjects provided written consent before participation. The study was carried out in accordance with the principles of the Declaration of Helsinki as revised in 2000.

As we did not directly perform any experiments on human samples and solely used publicly available data, we deemed it unnecessary to seek additional ethical approval for our study.

### Data

We searched transcriptome databases for skeletal muscle data from healthy and T2DM individuals. We found the most comprehensive dataset from the dbGaP database with the accession code phs001068.v1.p1. The gene expression data were obtained from vastus lateralis muscle using high-throughput RNA-Seq technology. The data comprise 91 healthy and 63 newly diagnosed subjects [7]. The transcriptome data were downloaded and were used for our analyses.

For validation of metabolite markers, the muscle gene expression data of healthy and diabetic subjects were taken from the GEO repository with the accession numbers GSE63887 and GSE81965 [8]. This data contains RNA-Seq samples from 24 individuals in four equal following groups: normal glucose tolerant (NGT)/non-obese, NGT/obese, T2DM/non-obese, and T2DM/obese.

### Muscle-specific metabolic model reconstruction

The muscle-specific metabolic model was reconstructed by integration of gene expression data to the generic model. Here, we used the HMR2 model as the generic model [9, 10]. The gene-protein-reaction (GPR) relationships in HMR2 were corrected using the Metabolic Atlas database [11] and BiGG model [12]. Moreover, the myocyte biomass reaction from the Bordbar muscle metabolic model was added to our model [4]. Muscle gene expression profiles of healthy individuals [7] were used. For the preprocessing, low expressed genes (< 5 read counts in less than 25 percentages of samples) were filtered and between-sample normalization according to the DESeq2 normalization method with gene length adjustment and log2 (gene expression+1) transformation were applied [13]. E-Flux algorithm [14] was employed to integrate gene-expression data to the HMR2 model and to reconstruct the context-specific metabolic model. To build a fully functional model we removed dead-end reactions.

### Constraint-based personalized metabolic modeling

Personalized GEMs were reconstructed by integrating gene expression data from each individual into the muscle-specific metabolic model. Pre-processed gene expression data and E-Flux algorithm were used here. Media conditions were set by body fluid metabolites [15]. We used maximizing flux through the production of mitochondrial ATP as the objective function. In addition, to ensure that the simulated cell is biologically relevant and functional,we set the lower bound of biomass reaction to 80% of the maximum biomass production by the healthy model [16]. For personalized metabolism simulation, flux variability analysis was applied. FVA is a common constraint-based and linear programming method to find the possible range of flux through each reaction while a set of constraints are satisfied [17]. The minimum (min) and maximum (max) possible fluxes through each reaction in each personalized model were obtained using FVA in Cobra Toolbox [18]. The glpk has been used for solving the linear programming problem. To find perturbed reactions in the T2DM state, the two-sample t-test was applied on the min and max fluxes between healthy and T2DM groups. The Benjamini–Hochberg procedure was used to apply multiple testing corrections and reactions with false discovery rates less than 0.1 were regarded as significant perturbed reactions.

### Enrichment analyses

To looks for significantly dysregulated pathways and metabolites, enrichment analyses were performed. Significant perturbed reactions were used as the reaction set to be enriched. Pathway-reaction set and metabolite-reaction sets were created using our reconstructed muscle metabolic model. Metabolites were assigned to the reaction if it participated in the corresponding reaction as either substrate or products. For enrichment analysis, hypergeometric 1-sided test with FDR correction for multiple testing correction was used.

### Metabolite-centric network

Metabolite-centric is one of the most common representations of the metabolic network. Metabolites are considered as the nodes of the network. Then two nodes are linked if both of them participate in the reaction, one as the substrate and the other as the product. We reconstructed the metabolite-centric network from perturbed reactions. For simplicity, transport and exchange reactions were excluded and unique metabolites instead of the compartmentalized ones were used. We also excluded the currency metabolites. To find the significant connected components of dysregulated metabolites, metabolites with an adjusted p-value greater than 0.05 were filtered. Adjusted p-values were obtained from metabolite enrichment analysis. The metabolite-centric subnetwork was visualized using Cytoscape version 3.8.2 [19].

### Classification and feature selection

An SVM classifier with a polynomial kernel of order 2 was used for classification. The classifier was evaluated by 10-fold stratified cross-validation and analysis of accuracy, sensitivity, and specificity were reported.


$$Accuracy=\frac{TP+TN}{TP+TN+FP+FN}$$

$$Sensitivity=\frac{TP}{TP+FN}$$

$$Specificity=\frac{TN}{TN+FP}$$

True Positives (TP) refers to the T2DM individuals that are correctly classified as patients. False Positives (FP) refers to the healthy individuals that are incorrectly classified as patients. True Negatives (TN) is the number of healthy individuals that correctly classified as healthy, and False Negatives (FN) is the number of T2DM individuals that are incorrectly classified as healthy.

For feature selection, a feature selection method, based on a hybrid of genetic algorithm (GA) and support vector machine was employed. In the GA, the feature subset is encoded as the candidate solution on a chromosome-like structure. The population is formed from a set of chromosomes that mutation and crossing over in it occur to generate the next generation. A fitness score is calculated for each chromosome representing adaptation of it to the environment and better feature subsets have more chance of reproducing the next generation. Creating the next generations will be continued until a stopping criterion is satisfied.

Here, a binary GA was employed that the binary values of 1 and 0 represent the presence or absence of a specific feature at the particular chromosome, respectively. The chromosome length was set to the number of features. The population size and the maximal number of generations were set to 300 chromosomes and 100 generations, respectively. We used the accuracy of the SVM classifier as the fitness score. The GA was stopped if the fitness score was reached an accuracy higher than 90 percent or the maximum number of generations was reproduced.

## Results

### Comprehensive high-throughput RNA-Seq data from skeletal muscle

We looked for the most comprehensive skeletal muscle gene expression profiles. We queried the transcriptome repositories for individuals’ expression profiles with the following criteria: 1) newly diagnosed with T2DM. 2) Taking no medication for diabetes. 3) Do not have any other disease affecting the analysis. This resulted in data with the accession code phs001068.v1.p1 in the dbGaP database. Diabetic patients in this sample have been newly diagnosed as T2DM patients, and in most of them, the fasting blood glucose concentration was around seven mmol/l. Table 1 shows the characteristics of the subjects of the samples used in this study. Investigation of metabolism reprogramming in these patients who are at the early stages of T2DM has significant value in identifying the underlying disorders in T2DM. We used gene expression data of healthy individuals in this sample to reconstruct the muscle-specific metabolic model.

**Table 1:** Subjects characteristics of the individuals in the dataset [7]. Means ± standard deviation values for fasting plasma glucose, fasting serum insulin, body mass index (BMI), and waist/hip ratio (WHR) in each diabetic and healthy group are shown.

Sample	Glucose (mmol/L)	Insulin (mu/l)	BMI	WHR
**T2DM**	7.17 ± 0.66	10.68 ± 6.94	29.37 ± 5.04	0.99 ± 0.07
**Healthy**	5.62 ± 0.33	6.87 ± 3.34	26.35 ± 3.47	0.92 ± 0.08

### Human muscle-specific metabolic model reconstruction

Reconstruction of the muscle-specific metabolic model requires the human generic metabolic model as the template. Here, the Human Metabolic Reaction 2 (HMR2) [10] was employed as the template metabolic model. The gene expression data are integrated into the model using GPR relationships. Proteins/protein complexes are linked to the corresponding genes by GPRs that are typically described with Boolean rules. An ’AND’ logic operator between two genes implies the required expression of both of them for a protein to have a function; an ‘OR’ operator denotes any of the related genes that can produce protein. In the HMR2 model, however, each reaction is assigned to the corresponding genes but the Boolean operators were all OR annotated. Since the right GPR rules are crucial for metabolic simulation, we manually curated them using the Metabolic Atlas database [11] and BiGG models [12]. The updated HMR2 model was used for subsequent steps.

The biomass reaction is used in metabolic models to represent all metabolites required for cell growth and maintenance. We included the biomass function in the template model to ensure that the simulated fluxes also assert muscle maintenance and viability. The muscle-specific biomass maintenance function was built using the information obtained from the experimental measurements and literature [4]. The process of formulating muscle-specific biomass functions has been described in their publication [4]. The biomass reaction was constructed and added to the template model.

The process of reconstructing the muscle-specific metabolic model requires the expression data to infer active reactions from the template model. The preprocessed transcriptome data from the healthy group were used to integrate into the model. E-flux algorithm [14] was applied to build the muscle-specific metabolic model. Reactions with dead-end metabolites were detected and removed from the model to have a fully functional model. Dead-end metabolites are those that are only consumed or produced within the network. Reactions with dead-end metabolites can not carry flux and are blocked. The final model comprises 3560 metabolites, 5714 reactions and, 2704 genes. Table 2 shows the characteristics of our muscle-specific metabolic model in comparison with HMR2. The reconstructed muscle metabolic model in this study is freely available at https://github.com/Maryamkhn/Muscle_MetNet_CBB.

**Table 2:** Comparison of HMR2 metabolic model with reconstructed muscle-specific metabolic model in the present study.

	HMR2 model	Muscle model
**Reactions**	8181	5714
**Metabolites**	6006	3560
**Genes**	3765	2704

For quality controls and validation, we first checked for biomass production. This asserts that all the required reactions to having an alive model are satisfied. Then, 56 metabolic tasks known to occur in all types of human cells were checked; Important pathways including glycolysis, TCA cycle, pentose phosphate, cellular respiration, synthesis of nucleotides and lipids, oxidation of fatty acids were checked. The complete list of these metabolic tasks can be found in [20]. Moreover, for muscle-specific validation, same as Bordbar et.al. [4], we tested the ability of the model to produce ATP from several different sources like glucose, fatty acids, glycogen, branched-chain amino acids, and ketone bodies. The model successfully passed all these tests. The reconstructed muscle-specific metabolic model was employed for further analyses.

### Metabolic reprogramming in the muscle at the early stage of T2DM

The reconstructed muscle metabolic model can be employed as a context to investigate metabolic reprogramming in diabetic muscles. Integrating gene expression data with the metabolic model can unravel pathways implicated in the disease. Here, we applied constraint-based personalized metabolic modeling. We reconstructed personalized metabolic models for healthy and T2DM individuals. Then, using a constraint-based approach, metabolism was simulated at the personalized level. In this approach, the metabolic model is converted to the mathematical format, and several constraints (e.g. adjusting lower and upper bound of fluxes in each reaction) were imposed on the model. Combining high-throughput transcriptional data and constraint-based models provides an opportunity to infer cellular metabolism at the relevant physiological state [21]. Flux variability analysis (FVA) is a well-known constraint-based approach, which can provide allowable flux ranges in the metabolic model through linear programming based strategy [17].

**Fig 2:**
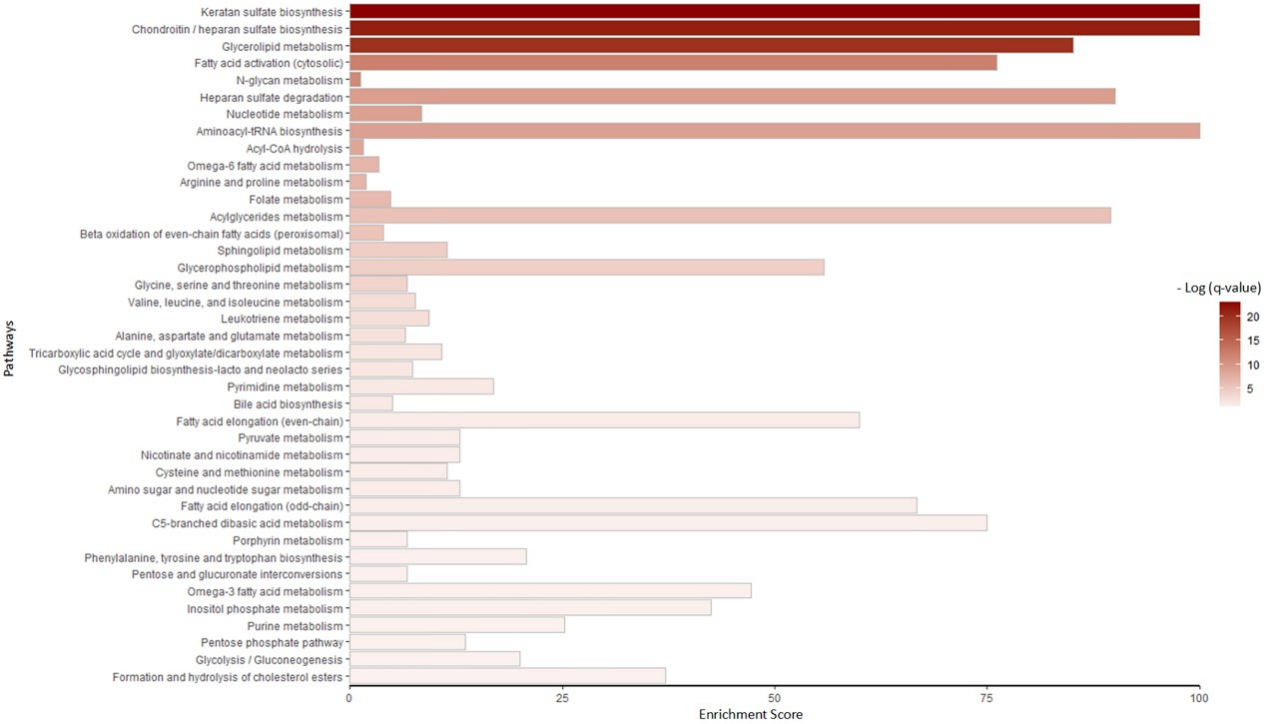
Pathway enrichment analysis. Perturbed reactions obtained from metabolic analysis in T2DM were used as reaction set to be enriched. The X-axis indicates the enrichment score. This score is calculated as the percentages of the perturbed reactions in each pathway divided by the total number of reactions in the corresponding pathway. Colors also show adjusted p-value obtained from pathway enrichment analysis.

**Fig 3:**
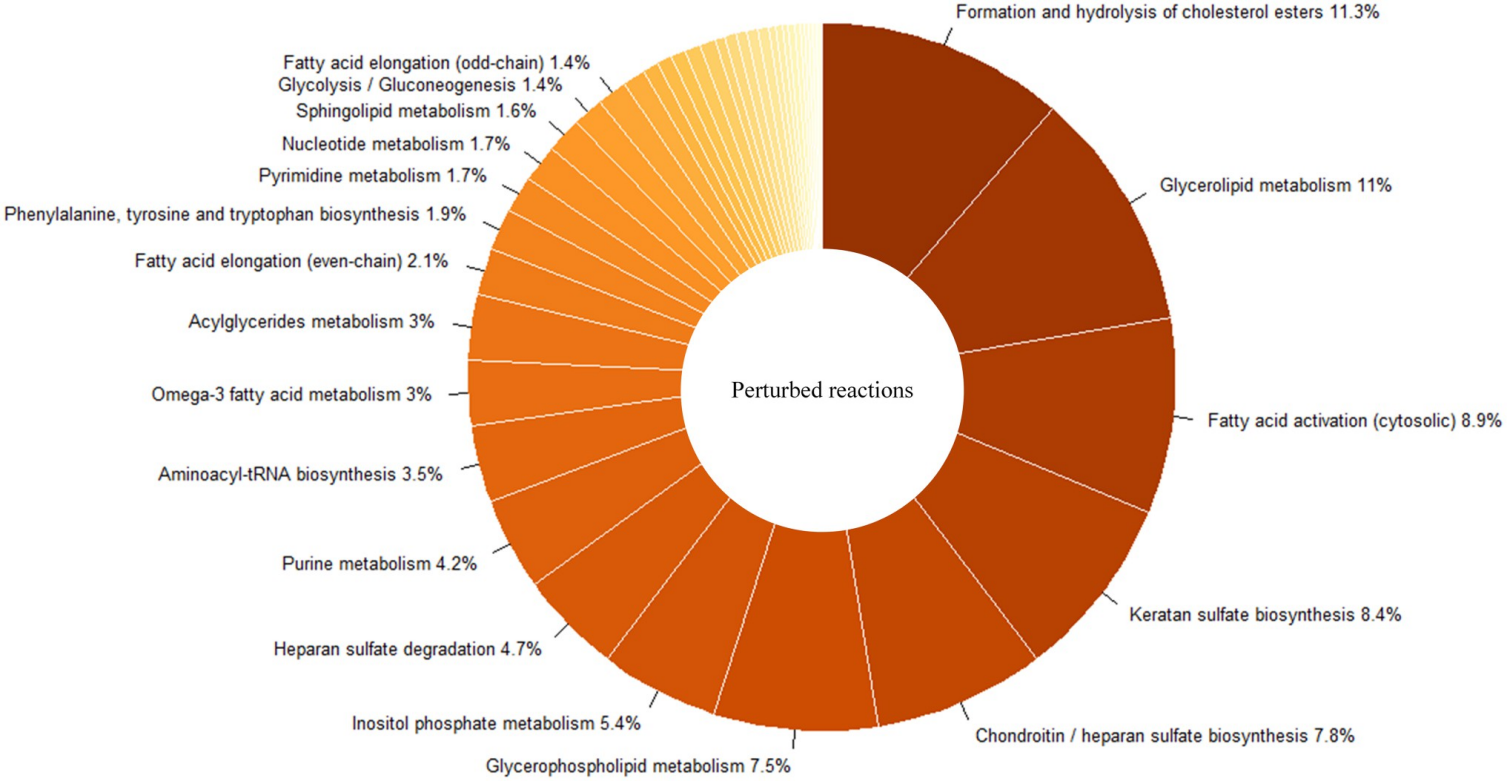
The distribution of perturbed reactions in enriched pathways. These perturbed reactions obtained from metabolic analysis in T2DM. Numbers indicate the percentage of reactions that belong to each pathway.

This analysis resulted in perturbed reactions involving in several pathways. The perturbations in the glucose to glucose-6-phosphate conversion reaction and the glucose exchange reaction were also observed. Many reactions contributing to fatty acids and lipid metabolism were dysregulated. Also, changes in fluxes of several reactions implicated in inositol phosphate, chondroitin, and heparin sulfate metabolism were observed. To find statistically significant enriched pathways, pathway enrichment analysis was employed. Fig 2 shows enrichment scores of these significant pathways. This score is calculated as the percentages of the perturbed reactions in each pathway divided by the total number of reactions in the corresponding pathway. For simplicity, carnitine shuttles, transport, and exchange reactions are not shown. Keratan sulfate, chondroitin/heparan sulfate biosynthesis, and glycerolipid metabolism have the most enrichment scores. Moreover, the distribution of perturbed reactions in these significant pathways is shown in Fig 3. Formation and hydrolysis of cholesterol esters, glycerolipid metabolism, fatty acid activation, keratan sulfate biosynthesis, chondroitin/heparan sulfate biosynthesis, glycerophospholipid metabolism, and inositol phosphate metabolism are the top enriched pathways in this figure.

**Fig 4:**
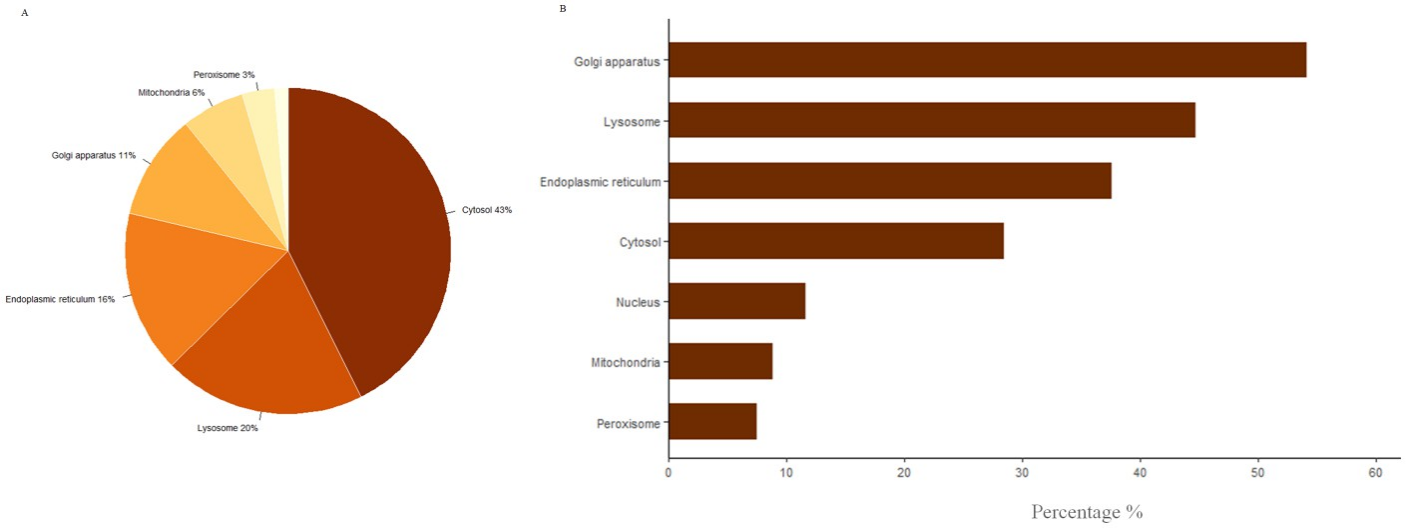
Dysmetabolism at the subcellular level. A) Distribution of perturbed reactions based on the subcellular compartments. B) The percentage of perturbed reactions in each compartment.

To reveal metabolic alterations at the subcellular compartment level, we classified each perturbed reaction based on the compartment the reaction occurs inside it ((Fig 4A). As is shown, cytosol, lysosome, and endoplasmic reticulum are the top dysregulated compartments. Cytosol due to the high number of cytosolic reactions has the largest amount of perturbed reaction. To understand which compartment is the most dysregulated ones we adjusted perturbed reactions in each compartment by the total number of reactions in the corresponding compartment (transport reactions were excluded). Fig 4B shows the result of this analysis. This figure demonstrates that Golgi, lysosome, and endoplasmic reticulum were mostly affected. Golgi has mainly encountered dysmetabolism in chondroitin, heparin, and keratan biosynthesis. Perturb reactions implicated in the formation and hydrolysis of cholesterol esters, heparan sulfate degradation, and chondroitin sulfate degradation comprise the major dysmetabolism in the lysosome. In addition, the endoplasmic reticulum is mostly affected by perturbation in the carnitine shuttle subsystem.

**Fig 5:**
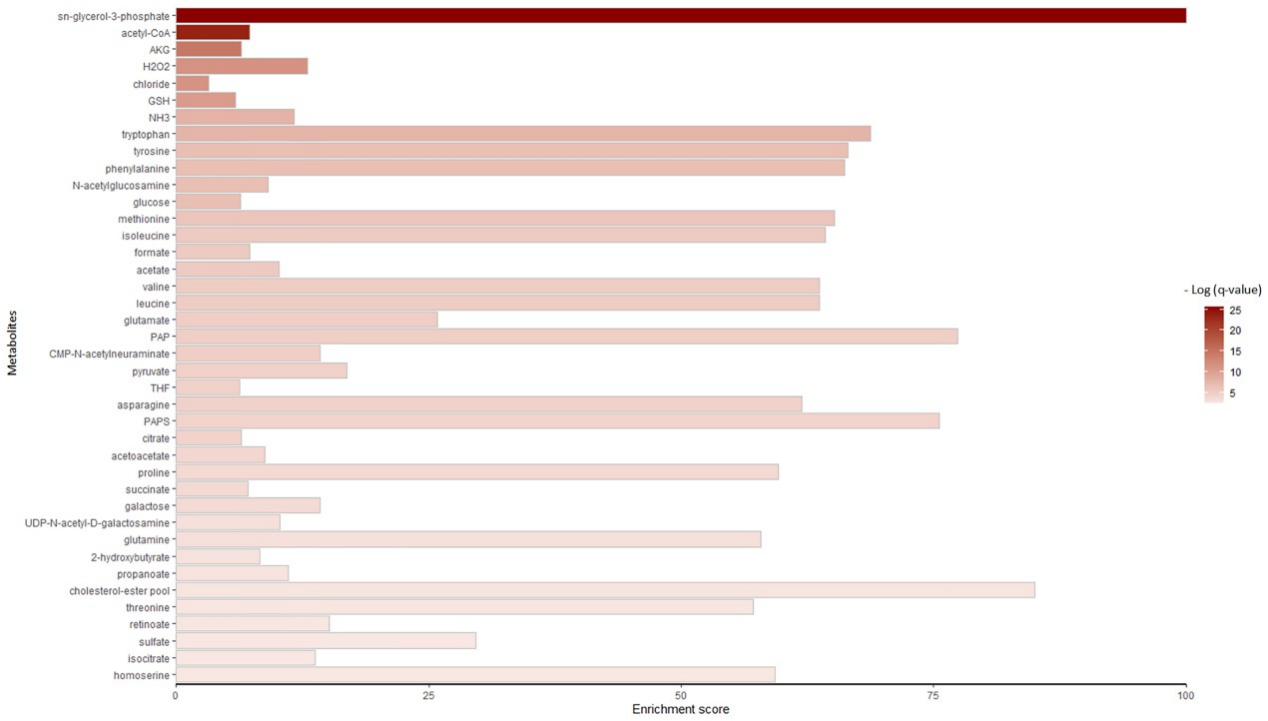
Metabolites enrichment analysis. The X-axis indicates the enrichment score. This score is calculated as the percentage of perturbed reactions to the total number of reactions in which the corresponding metabolite involved. Colors also show adjusted p-values obtained from enrichment analysis.

We also provided enrichment analysis of significant perturbed reactions in the context of metabolites. Several important metabolites mainly implicated in amino acids, fatty acids, and lipid metabolism were enriched; Metabolites including glucose, mitochondrial ATP, CoA, fatty acids, activated fatty acids, cholesterol, amino acids, pyruvate, AKG, succinate, homoserine, NADH, FADH, glutathione, Acetyl-CoA, CMP-N-acetylneuraminate, THF, 5,10-methenyl-THF, and 10-formyl-THF. Fig 5 shows the top 40 significant enriched metabolites with corresponding enrichment scores (currency metabolites are excluded). The complete list of the enriched metabolites in unique and compartmentalized form can be found in Additional File 1, S1 and S2 Tables in S1 File.

**Fig 6:**
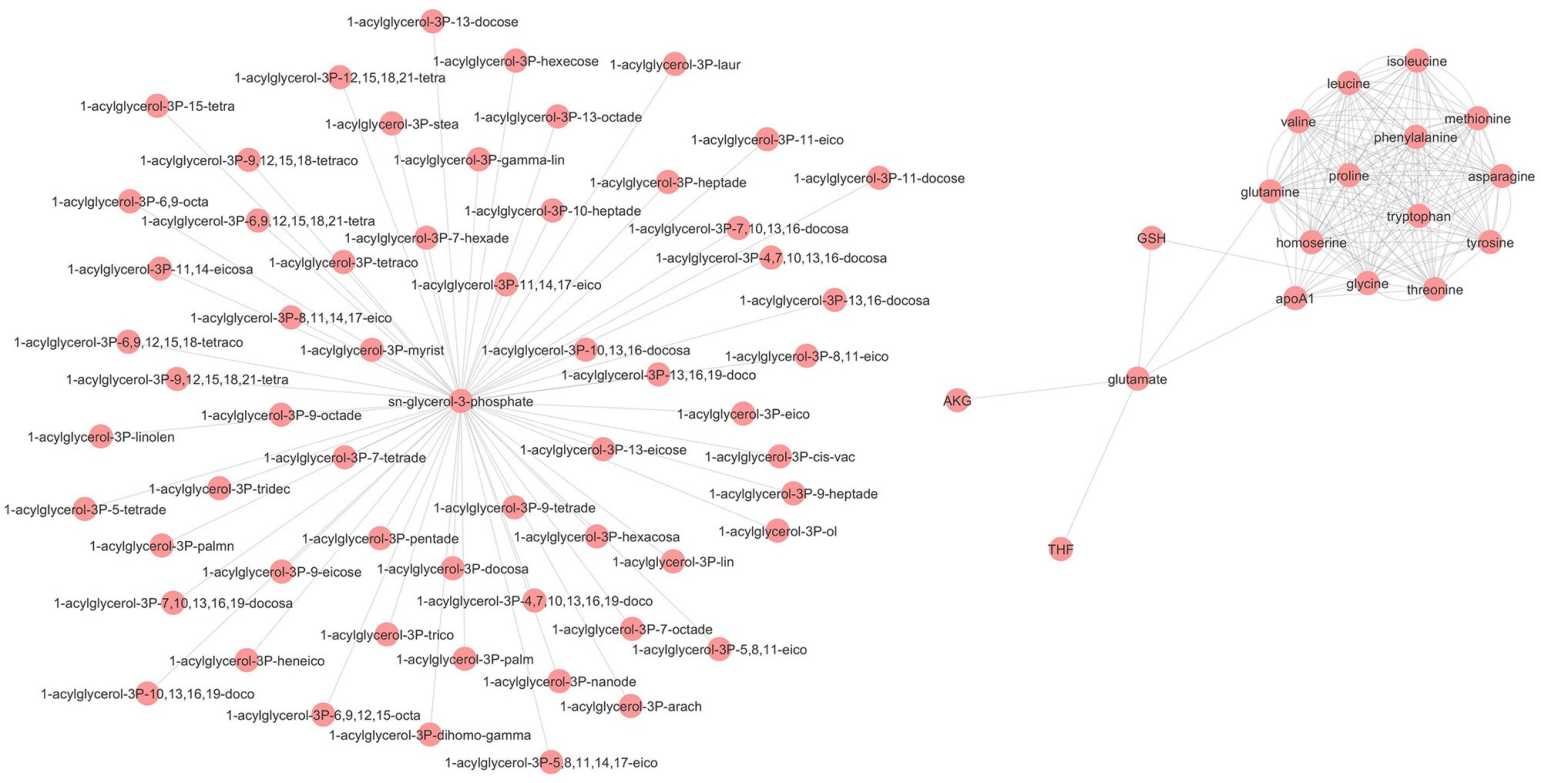
Connected components of significantly dysregulated metabolites. The metabolite-centric network was reconstructed from the perturbed reactions in T2DM. Insignificant enriched metabolites were removed from the network. The two biggest connected components were found. The right component mainly consists of amino acids. The left component comprises metabolites involved in the glycerolipid metabolism.

We constructed a metabolite-centric network from the perturbed metabolic network to find the connected component of significantly dysregulated metabolites. Enriched metabolites with adjusted p-value lower than 0.05 were preserved and other metabolites were removed from the metabolite-centric network. We used Cytoscape biological network tool [19] to visualize the subnetwork. Fig 6 shows the two biggest connected components as significantly dysregulated ones; One is related to the glycerolipid metabolism and the other related to the metabolism of the amino acids. We also found tetrahydrofolate (THF) and glutathione (GSH) connected to the amino acids related component.

### Potential metabolite markers prediction

The genome‐scale model of human metabolism provides an opportunity to predict potential biofluid markers associated with the diseases. The overall approach involves identifying exchange metabolites that their flux range in disease state is shifted compared to healthy ones. The successful performance of this method has already been evaluated and validated [22]. Here, we applied this method to find metabolite markers of insulin resistance in muscle. Two generic metabolic models of healthy and diabetic muscles were reconstructed using average gene expression data of each group and the E-Flux method; Then, FVA analysis was applied to them. The exchange reactions in which the flux range was shifted relative to the normal state were selected. This approach resulted in 32 exchange reactions. In order to evaluate the discriminative capability of these reactions at the individual level, a machine learning approach was employed. To do this, the generated minimum and maximum fluxes of these reactions obtained from personalized FVA analysis were used as the features for classification with SVM. This evaluation resulted in 72.22% accuracy, 81.11% specificity, and 60.32% sensitivity. We found that these exchange metabolites altered at the individuals’ level.

**Fig 7:**
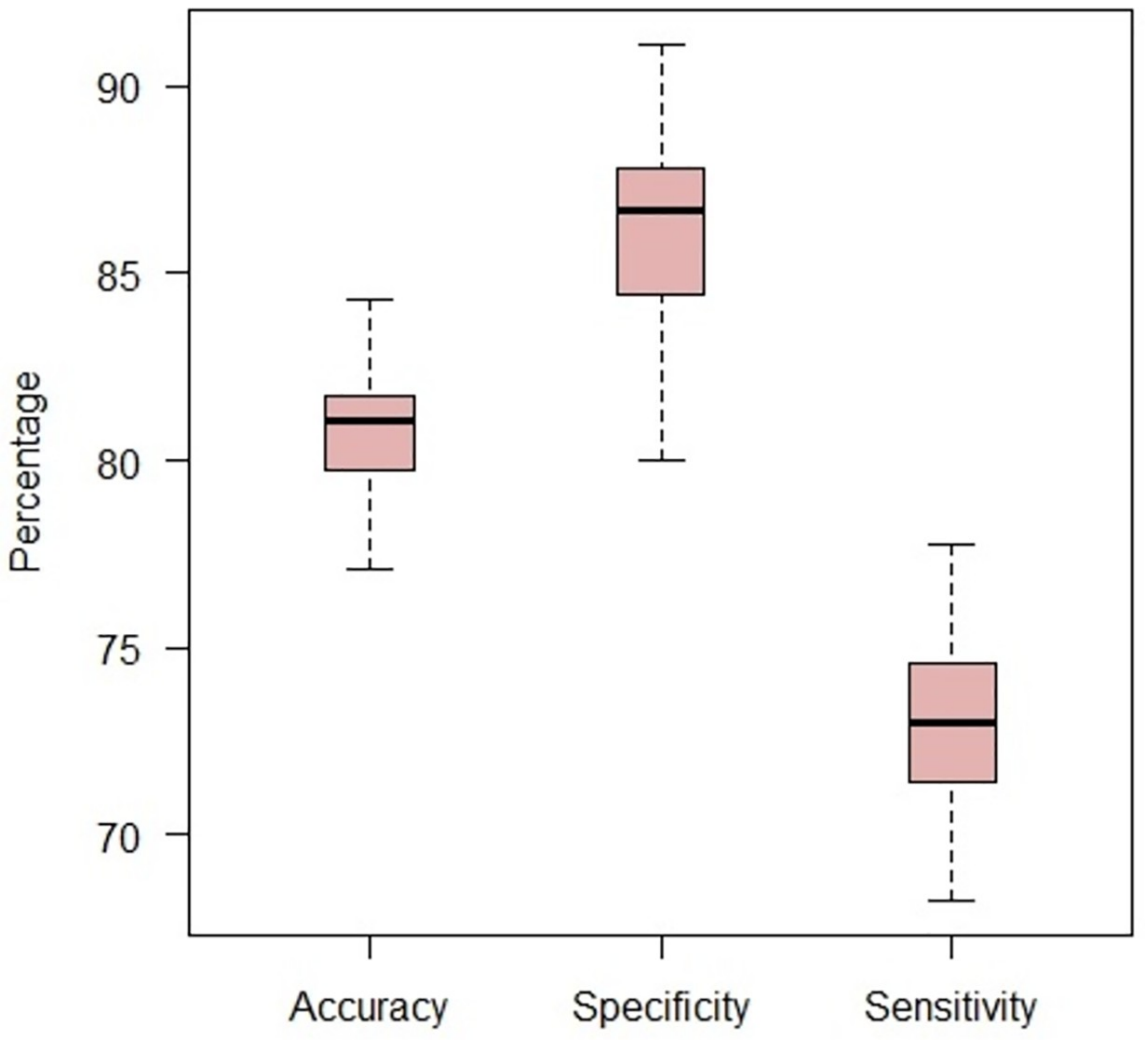
Boxplot of SVM evaluation using 13 metabolite markers as features. 10-fold cross-validation was repeated 100 times and the SVM performance was evaluated.

To achieve a fewer and better feature subset with improvement in classification accuracy, a wrapper feature selection method was employed that comprises a combination of GA and SVM as the feature selector and classifier, respectively. Several subsets of features with which the SVM classifier can discriminate diabetic patients from healthy ones with approximately 90 percentages accuracy were found. This GA-SVM procedure was iterated 100 times resulting in 100 feature subsets; then, the observation frequency of each feature in these iterations was calculated. These frequencies were considered as indicative of the importance of features and features with at least 80% frequency regarded as top-ranked features. The top-ranked features were related to 13 reactions exchanging glucose, alanine, aspartate, sodium, hyaluronate, galactose, GQ1b, acetaldehyde, deoxyribose, 4-OH-Estradiol, hypoxanthine, retinoate, and methylglyoxal. Subsequently, the SVM classification performance with the top-ranked features was assessed. Our analysis revealed that using these top-ranked features improved classification accuracy to 81.04% with 73.02% sensitivity and 86.67% specificity. Since the classifier performance will vary depending on which samples are assigned to the training set and which ones to the test set, we repeated the 10-fold cross-validation 100 times and evaluated the SVM performance. The boxplot of this evaluation is shown in Fig 7.

For further validation, we assessed the performance of these top-ranked features with data obtained from another study [8]. This data comprises RNA-Seq samples from the vastus lateralis muscle of 24 participants, divided into four following groups: normal glucose tolerant (NGT)/non-obese, NGT/obese, T2DM/non-obese, and T2DM/obese. After preprocessing of the read counts, personalized metabolic models were reconstructed and FVA analysis was performed. Associated fluxes of top-ranked features were obtained and were used as the features for the SVM classifier.

We divided these people into NGT and diabetic groups regardless of the obesity state and the generated fluxes of marker reactions in these 24 participants were used for validation with SVM. This analysis resulted in 49.46% accuracy, 42.25% specificity, and 56.67% sensitivity, which is remarkably low, probably due to the presence of normoglycemic obese people in the healthy group. Although the blood glucose level in these individuals is in the normal range, high levels of fasting blood insulin level (60 ± 33 pmol/l in NGT/obese vs 25 ±7 pmol/l in NGT/non-obese) indicates insulin resistance in this group, which is compensated by more insulin secretion. Thus, we decided to remove data from NGT/obese individuals and re-evaluated markers in this set. The result significantly altered with 78.50% accuracy, 70.17% specificity, and 82.67% sensitivity. Therefore, the metabolites markers are related to insulin-resistant metabolism and can successfully discriminate insulin-resistant individuals from healthy ones.

## Discussions

Here, we aimed to provide a holistic view of the metabolism remodeling in skeletal muscle of newly diagnosed T2DM patients. Since diabetes is a progressive metabolic disease, our goal was to identify the metabolic alterations at the early stages of the disease. We used the gene expression data from the most comprehensive human skeletal muscle transcriptome study to date. The diabetic sample consists of participants who have newly been diagnosed with diabetes (with an average blood glucose concentration of ~ 7.2 mmol/L) and had not taken any medications. For understanding metabolism alterations in T2DM, we first reconstructed our functional muscle-specific metabolic model. We could not utilize Bordbar [4] or Nogiec [5] muscle metabolic models due to them being small-scale. Varemo model [6] was a complete muscle metabolic model, but because of the wrong GPRs, it was not suitable for simulation. Thus, we reconstructed our comprehensive skeletal muscle metabolic model. The model quality was successfully validated. This model is freely available and can be used for other muscle metabolic researches.

We employed the reconstructed model for system-level investigation of dysmetabolism in T2DM. Personalized metabolic models with constraint-based simulations were applied to quantitatively compare metabolic capabilities between healthy and newly diagnosed diabetic patients. Perturbed reactions and subsequently affected pathways and metabolites were identified. We found that significant metabolic alterations occurred in several pathways. Pathways implicated in membrane and ECM biosynthesis were dysregulated. Perturbations in the metabolism of keratin sulfate, chondroitin sulfate, and heparan sulfate, inositol phosphate, glycerolipid, glycerophospholipid, and sphingolipid metabolism were observed. Chondroitin sulfate, keratin sulfate, heparan sulfate, and hyaluronic acid are glycosaminoglycans (GAGs). GAGs play a vital role in cell physiology including cell signaling, proliferation, and cell adhesion. The insulin-sensitizing and anti-diabetic impacts of some GAGs have been reported [23, 24]. Perturbations in GAGs related pathways and sphingolipid metabolism imply the role of the ECM in insulin resistance, which is involved in the regulation of insulin action. Inositol mediates insulin signal transduction, is associated with glucose uptake, and plays a vital role in oxidative stress and inflammation. Administration of inositol supplements improves glucose metabolism and insulin resistance [25].

Significant alterations in lipid metabolism including glycerolipid, glycerophospholipid, sphingolipid, and glycosphingolipid metabolism were found in our analyses. These pathways mediate the production of major constituents of the plasma membrane. These metabolites act as structural components of membranes and as signaling molecules. Dysfunctional lipid metabolism can lead to membrane rearrangement, changing its integrity, and interrupt cell signaling. Perturbation in these pathways also triggers feedback loops resulting in mitochondrial dysfunction, reactive oxygen species production, and inflammation. Studies have found several intermediates in these pathways implicated in insulin resistance [26].

Enrichment analysis also revealed perturbation in amino acids. Previous studies have demonstrated associations between amino acids and T2DM. In particular, the various pieces of evidence have implied the implication of branched-chain amino acids (BCAAs) in diabetes. Studies have shown that dysregulation of BCAAs metabolism results in the serine phosphorylation of insulin receptor substrates and subsequent uncoupling of insulin signaling [27]. Here, in this analysis BCAAs and all other amino acids were affected by the metabolic reprogramming in T2DM. Since amino acids also circulate in blood plasma, dysmetabolism can possibly be reflected in serum. In recent years, amino acids have been considered as a source of prognostic markers in T2DM [28]. Here, dysregulation of amino acids in the early stages of T2DM was observed which can support the significance of these metabolites in the early diagnosis of the disease. Also, tetrahydrofolic acid is connected to the amino acids in the metabolite-centric network. THF is a cofactor in the anabolism of amino acids. THF and its derivatives also contribute to purine, pyrimidine and nucleotide synthesis, and DNA methylation [29]. Alteration in folate metabolism can disrupt downstream processes. Limited research regarding the role of folate and its supplementation has been performed. Folate intakes have shown an inverse association with the incidence of T2DM [30–33]. It improves insulin resistance by modulating DNA methylation of genes associated with obesity and insulin resistance [34]. The role of folate metabolism in muscle insulin resistance could be the subject of further empirical researches.

Analysis of metabolic alteration at the sub-cellular level revealed that Golgi is mainly affected by metabolic remodeling in T2DM. This compartment implicates in building ECM metabolites. Many studies have been performed on the role of mitochondria and endoplasmic reticulum in the development of insulin resistance [35]; whereas, our knowledge about the role of Golgi is very limited. Based on the observations in the present study, we suppose the role of Golgi in insulin resistance should be further investigated.

Taken together, the main dysmetabolism was observed in ECM components, lipids, and amino acids related pathways. Muscle dysmetabolism changes the abundance of metabolites involved in the membrane and ECM. This metabolic reprogramming can interrupt the process of sensing insulin and transmitting signals into the cell. Decreased insulin sensitivity results in lower expression of insulin-responsive genes, reduced glucose uptake, and consequently changes in energy, glucose, fatty acids, and amino acid metabolisms.

As the final analysis, potential metabolite markers were predicted. Our approach led to the identification of 13 exchange metabolites that could discriminate healthy individuals from T2DM patients with 81% accuracy. We validated these markers using separate gene expression data from the Varemo et.al. study [8]. They have shown that muscle transcriptional reprogramming in obesity is similar to that which occur in T2DM [8]. Here, we found that using all data from this study, including normoglycemic obese individuals for validation, the accuracy of our proposed marker was notably low (~ 50%). We thought this low accuracy may be due to the metabolic similarities between obese and diabetic individuals. In fact, as noted in the Varemo et.al. study, obese and diabetic individuals have shown similar gene expression patterns [8] that can lead to similar metabolisms. Moreover, obese individuals had high levels of fasting blood insulin, which demonstrate insulin resistance in this group. To test this issue, we removed obese healthy individuals from the validation set. This improved accuracy to 78.50%. Therefore, this analysis confirmed both the Varemo et.al. claim about the similarity of the gene expression pattern between obese and diabetic individuals and the appropriate efficiency of our proposed markers in identifying insulin-resistant individuals. Although it is reasonable to assume that our model predicts insulin resistance biomarkers rather than obesity-related metabolic changes based on the fact that the diabetic samples in the test group included both non-obese and obese individuals, further research is needed to confirm that these biomarkers do not overlap with metabolic changes related to obesity. To achieve this, muscle gene expression data is required from individuals with normal blood sugar and insulin levels in both obese and non-obese groups, without drug use or confounding factors. Important metabolites such as methylglyoxal, hyaluronan, retinoic acid (vitamin A), sodium, alanine, and aspartate are present in the predicted markers. Notably, this method also successfully identified glucose as one of the markers. Methylglyoxal, a glycolytic by-product, is a toxic and highly reactive compound involved in cellular dysregulations. This compound modifies nucleotides, proteins, and lipids producing advanced glycation end products, contributing to diabetes complications. Methylglyoxal is associated with oxidative stress, cellular inflammation, and age-related disease such as diabetes [36]. Several studies have revealed the impact of methylglyoxal on insulin signaling pathways and insulin resistance [37, 38] and recently this metabolite has been introduced as an emerging marker for T2DM diagnosis [36]. Hyaluronan is an anionic GAG metabolite implicated in several functions like cell signaling, proliferation and migration, and angiogenesis. This metabolite also contributes to the inflammation and pathogenesis of T2DM [39, 40]. Studies have demonstrated that hyaluronan increases in the serum and skeletal muscle of T2DM subjects [41, 42]. Several analyses have shown the possible roles of vitamin A in glucose metabolism, and the progression of insulin resistance [43–45]. Association of purine metabolites such as xanthine and hypoxanthine with the risk of T2DM incidence and complications has been reported [46, 47]. Also, change in serum concentration of amino acids [48], and sodium [49] is associated with obesity and T2DM. In addition, the implication of gangliosides in insulin resistance has been shown [50–52]. In our study, GQ1b ganglioside was predicted in the top-ranked metabolites list that can be considered for future analysis. We also checked metabolomics-based studies of T2DM and the Human Metabolome Database for these metabolites [53]. We found that glucose, hypoxanthine, alanine, aspartate, galactose, hyaluronate, and methylglyoxal levels have been reported to be associated with T2DM [46, 53, 54]. This evidence confirms the efficiency of our method for identifying metabolite markers. These metabolite markers can be used for a further empirical investigation to verify their prognostic and diagnostic values in insulin resistance.

## Supporting information

S1 File The complete list of the enriched metabolites in the unique and compartmentalized forms obtained from metabolite enrichment analysis of perturbed reactions is provided in S1 and S2 Tables, respectively. (XLSX)

## Abbreviations

BCAA: Branched-chain amino acid
ECM: Extracellular matrix
FVA: Flux variability analysis
GA: Genetic algorithm
GAGs: Glycosaminoglycans
GEM: Genome-scale metabolic model
GPR: Gene-protein-reaction
HMR2: Human Metabolic Reaction 2
NGT: Normal glucose toleran
SVM: Support vector machine
T2DM: Type 2 diabetes mellitus
THF: Tetrahydrofolate

## References

[1] Saeedi P, Petersohn I, Salpea P, Malanda B, Karuranga S, Unwin N, et al. Global and regional diabetes prevalence estimates for 2019 and projections for 2030 and 2045: Results from the International Diabetes Federation Diabetes Atlas. Diabetes research and clinical practice. 2019;157:107843. 31518657 10.1016/j.diabres.2019.107843

[2] Stump CS, Henriksen EJ, Wei Y, Sowers JR. The metabolic syndrome: role of skeletal muscle metabolism. Annals of medicine. 2006;38(6):389–402. doi: 10.1080/07853890600888413 17008303

[3] Merz KE, Thurmond DC. Role of Skeletal Muscle in Insulin Resistance and Glucose Uptake. Compr Physiol. 2020;10(3):785–809. doi: 10.1002/cphy.c190029 32940941 PMC8074531

[4] Bordbar A, Feist AM, Usaite-Black R, Woodcock J, Palsson BO, Famili I. A multi-tissue type genome-scale metabolic network for analysis of whole-body systems physiology. BMC systems biology. 2011;5(1):180. doi: 10.1186/1752-0509-5-180 22041191 PMC3219569

[5] Nogiec C, Burkart A, Dreyfuss JM, Lerin C, Kasif S, Patti M-E. Metabolic modeling of muscle metabolism identifies key reactions linked to insulin resistance phenotypes. Molecular metabolism. 2015;4(3):151–63. doi: 10.1016/j.molmet.2014.12.012 25737951 PMC4338313

[6] Väremo L, Scheele C, Broholm C, Mardinoglu A, Kampf C, Asplund A, et al. Proteome-and transcriptome-driven reconstruction of the human myocyte metabolic network and its use for identification of markers for diabetes. Cell reports. 2015;11(6):921–33. doi: 10.1016/j.celrep.2015.04.010 25937284

[7] Scott LJ, Erdos MR, Huyghe JR, Welch RP, Beck AT, Wolford BN, et al. The genetic regulatory signature of type 2 diabetes in human skeletal muscle. Nature communications. 2016;7. doi: 10.1038/ncomms11764 27353450 PMC4931250

[8] Väremo L, Henriksen TI, Scheele C, Broholm C, Pedersen M, Uhlén M, et al. Type 2 diabetes and obesity induce similar transcriptional reprogramming in human myocytes. Genome medicine. 2017;9(1):47. doi: 10.1186/s13073-017-0432-2 28545587 PMC5444103

[9] Mardinoglu A, Agren R, Kampf C, Asplund A, Nookaew I, Jacobson P, et al. Integration of clinical data with a genome‐scale metabolic model of the human adipocyte. Molecular systems biology. 2013;9(1). doi: 10.1038/msb.2013.5 23511207 PMC3619940

[10] Mardinoglu A, Agren R, Kampf C, Asplund A, Uhlen M, Nielsen J. Genome-scale metabolic modelling of hepatocytes reveals serine deficiency in patients with non-alcoholic fatty liver disease. Nature communications. 2014;5:3083. doi: 10.1038/ncomms4083 24419221

[11] Robinson JL, Kocabaş P, Wang H, Cholley P-E, Cook D, Nilsson A, et al. An atlas of human metabolism. Science Signaling. 2020;13(624). doi: 10.1126/scisignal.aaz1482 32209698 PMC7331181

[12] King ZA, Lu J, Dräger A, Miller P, Federowicz S, Lerman JA, et al. BiGG Models: A platform for integrating, standardizing and sharing genome-scale models. Nucleic acids research. 2016;44(D1):D515–D22. doi: 10.1093/nar/gkv1049 26476456 PMC4702785

[13] Love MI, Huber W, Anders S. Moderated estimation of fold change and dispersion for RNA-seq data with DESeq2. Genome biology. 2014;15(12):550. doi: 10.1186/s13059-014-0550-8 25516281 PMC4302049

[14] Colijn C, Brandes A, Zucker J, Lun DS, Weiner B, Farhat MR, et al. Interpreting expression data with metabolic flux models: predicting Mycobacterium tuberculosis mycolic acid production. PLoS Comput Biol. 2009;5(8):e1000489. doi: 10.1371/journal.pcbi.1000489 19714220 PMC2726785

[15] Hadi M, Marashi S-A. Reconstruction of a generic metabolic network model of cancer cells. Molecular BioSystems. 2014;10(11):3014–21. doi: 10.1039/c4mb00300d 25196995

[16] Chénard T, Guénard F, Vohl M-C, Carpentier A, Tchernof A, Najmanovich RJ. Remodeling adipose tissue through in silico modulation of fat storage for the prevention of type 2 diabetes. BMC systems biology. 2017;11(1):60. doi: 10.1186/s12918-017-0438-9 28606124 PMC5468946

[17] Mahadevan R, Schilling C. The effects of alternate optimal solutions in constraint-based genome-scale metabolic models. Metabolic engineering. 2003;5(4):264–76. doi: 10.1016/j.ymben.2003.09.002 14642354

[18] Heirendt L, Arreckx S, Pfau T, Mendoza SN, Richelle A, Heinken A, et al. Creation and analysis of biochemical constraint-based models: the COBRA Toolbox v3. 0. arXiv preprint arXiv:171004038. 2017. 10.1038/s41596-018-0098-2 PMC6635304 30787451

[19] Shannon P, Markiel A, Ozier O, Baliga NS, Wang JT, Ramage D, et al. Cytoscape: a software environment for integrated models of biomolecular interaction networks. Genome research. 2003;13(11):2498–504. doi: 10.1101/gr.1239303 14597658 PMC403769

[20] He Y, Wang Y, Zhang B, Li Y, Diao L, Lu L, et al. Revealing the metabolic characteristics of human embryonic stem cells by genome‐scale metabolic modeling. FEBS letters. 2018;592(22):3670–82. doi: 10.1002/1873-3468.13255 30223296

[21] Bordbar A, Monk JM, King ZA, Palsson BO. Constraint-based models predict metabolic and associated cellular functions. Nature Reviews Genetics. 2014;15(2):107. doi: 10.1038/nrg3643 24430943

[22] Shlomi T, Cabili MN, Ruppin E. Predicting metabolic biomarkers of human inborn errors of metabolism. Molecular systems biology. 2009;5(1). doi: 10.1038/msb.2009.22 19401675 PMC2683725

[23] Reynés B, Serrano A, Petrov PD, Ribot J, Chetrit C, Martínez-Puig D, et al. Anti-obesity and insulin-sensitising effects of a glycosaminoglycan mix. Journal of Functional Foods. 2016;26:350–62.

[24] Moto M, Takamizawa N, Shibuya T, Nakamura A, Katsuraya K, Iwasaki K, et al. Anti-diabetic effects of chondroitin sulfate on normal and type 2 diabetic mice. Journal of Functional Foods. 2018;40:336–40.

[25] Bevilacqua A, Bizzarri M. Inositols in insulin signaling and glucose metabolism. International journal of endocrinology. 2018;2018. doi: 10.1155/2018/1968450 30595691 PMC6286734

[26] Meikle PJ, Summers SA. Sphingolipids and phospholipids in insulin resistance and related metabolic disorders. Nature Reviews Endocrinology. 2017;13(2):79. doi: 10.1038/nrendo.2016.169 27767036

[27] Lynch CJ, Adams SH. Branched-chain amino acids in metabolic signalling and insulin resistance. Nature Reviews Endocrinology. 2014;10(12):723. doi: 10.1038/nrendo.2014.171 25287287 PMC4424797

[28] Hameed A, Mojsak P, Buczynska A, Suleria HAR, Kretowski A, Ciborowski M. Altered Metabolome of Lipids and Amino Acids Species: A Source of Early Signature Biomarkers of T2DM. Journal of Clinical Medicine. 2020;9(7):2257. doi: 10.3390/jcm9072257 32708684 PMC7409008

[29] Li J, Goh CE, Demmer RT, Whitcomb BW, Du P, Liu Z. Association between serum folate and insulin resistance among US nondiabetic adults. Scientific reports. 2017;7(1):1–7. 28835661 10.1038/s41598-017-09522-5 PMC5569086

[30] Pravenec M, Kožich V, Krijt J, Sokolová J, Zídek V, Landa V, et al. Folate deficiency is associated with oxidative stress, increased blood pressure, and insulin resistance in spontaneously hypertensive rats. American journal of hypertension. 2013;26(1):135–40. doi: 10.1093/ajh/hps015 23382337 PMC3626034

[31] Stewart CP, Christian P, Schulze KJ, LeClerq SC, West KP Jr, Khatry SK. Antenatal micronutrient supplementation reduces metabolic syndrome in 6-to 8-year-old children in rural Nepal. The Journal of nutrition. 2009;139(8):1575–81. doi: 10.3945/jn.109.106666 19549749

[32] Solini A, Santini E, Ferrannini E. Effect of short-term folic acid supplementation on insulin sensitivity and inflammatory markers in overweight subjects. International journal of obesity. 2006;30(8):1197–202. doi: 10.1038/sj.ijo.0803265 16491109

[33] Setola E, Monti LD, Galluccio E, Palloshi A, Fragasso G, Paroni R, et al. Insulin resistance and endothelial function are improved after folate and vitamin B12 therapy in patients with metabolic syndrome: relationship between homocysteine levels and hyperinsulinemia. European journal of endocrinology. 2004;151(4):483–90. doi: 10.1530/eje.0.1510483 15476449

[34] Li W, Tang R, Ma F, Ouyang S, Liu Z, Wu J. Folic acid supplementation alters the DNA methylation profile and improves insulin resistance in high-fat-diet-fed mice. The Journal of nutritional biochemistry. 2018;59:76–83. doi: 10.1016/j.jnutbio.2018.05.010 29986310

[35] Yaribeygi H, Farrokhi FR, Butler AE, Sahebkar A. Insulin resistance: Review of the underlying molecular mechanisms. Journal of cellular physiology. 2019;234(6):8152–61. doi: 10.1002/jcp.27603 30317615

[36] Bhat LR, Vedantham S, Krishnan UM, Rayappan JBB. Methylglyoxal–an emerging biomarker for diabetes mellitus diagnosis and its detection methods. Biosensors and bioelectronics. 2019. 10.1016/j.bios.2019.03.010 30921627

[37] Riboulet-Chavey A, Pierron A, Durand I, Murdaca J, Giudicelli J, Van Obberghen E. Methylglyoxal impairs the insulin signaling pathways independently of the formation of intracellular reactive oxygen species. Diabetes. 2006;55(5):1289–99. doi: 10.2337/db05-0857 16644685

[38] Maessen DE, Stehouwer CD, Schalkwijk CG. The role of methylglyoxal and the glyoxalase system in diabetes and other age-related diseases. Clinical Science. 2015;128(12):839–61. doi: 10.1042/CS20140683 25818485

[39] Litwiniuk M, Krejner A, Speyrer M, Gauto A, Grzela T. Hyaluronic acid in inflammation and tissue regeneration. Wounds. 2016;28(3):78–88. 26978861

[40] Sainio A, Takabe P, Oikari S, Salomäki-Myftari H, Koulu M, Söderström M, et al. Metformin decreases hyaluronan synthesis by vascular smooth muscle cells. Journal of Investigative Medicine. 2020;68(2):383–91. doi: 10.1136/jim-2019-001156 31672719 PMC7063400

[41] Kang L, Lantier L, Kennedy A, Bonner JS, Mayes WH, Bracy DP, et al. Hyaluronan accumulates with high-fat feeding and contributes to insulin resistance. Diabetes. 2013;62(6):1888–96. doi: 10.2337/db12-1502 23349492 PMC3661617

[42] Nagy N, Sunkari VG, Kaber G, Hasbun S, Lam DN, Speake C, et al. Hyaluronan levels are increased systemically in human type 2 but not type 1 diabetes independently of glycemic control. Matrix Biology. 2019;80:46–58. doi: 10.1016/j.matbio.2018.09.003 30196101 PMC6401354

[43] Beydoun MA, Chen X, Jha K, Beydoun HA, Zonderman AB, Canas JA. Carotenoids, vitamin A, and their association with the metabolic syndrome: A systematic review and meta-analysis. Nutrition reviews. 2019;77(1):32–45. doi: 10.1093/nutrit/nuy044 30202882 PMC6277204

[44] Blaner WS. Vitamin A signaling and homeostasis in obesity, diabetes, and metabolic disorders. Pharmacology & therapeutics. 2019. doi: 10.1016/j.pharmthera.2019.01.006 30703416 PMC6520171

[45] Kuang H, Wei C-h, Wang T, Eastep J, Li Y, Chen G. Vitamin A status affects weight gain and hepatic glucose metabolism in rats fed a high-fat diet. Biochemistry and Cell Biology. 2019;97(5):545–53. doi: 10.1139/bcb-2018-0284 30802138

[46] Papandreou C, Li J, Liang L, Bulló M, Zheng Y, Ruiz-Canela M, et al. Metabolites related to purine catabolism and risk of type 2 diabetes incidence; modifying effects of the TCF7L2-rs7903146 polymorphism. Scientific reports. 2019;9(1):1–11. 30814579 10.1038/s41598-019-39441-6 PMC6393542

[47] Varadaiah YGC, Sivanesan S, Nayak SB, Thirumalarao KR. Purine metabolites can indicate diabetes progression. Archives of Physiology and Biochemistry. 2019:1–5. doi: 10.1080/13813455.2019.1663219 31517540

[48] Gar C, Rottenkolber M, Prehn C, Adamski J, Seissler J, Lechner A. Serum and plasma amino acids as markers of prediabetes, insulin resistance, and incident diabetes. Critical reviews in clinical laboratory sciences. 2018;55(1):21–32. doi: 10.1080/10408363.2017.1414143 29239245

[49] Soltani S, Kolahdouz Mohammadi R, Shab-Bidar S, Vafa M, Salehi-Abargouei A. Sodium status and the metabolic syndrome: A systematic review and meta-analysis of observational studies. Critical reviews in food science and nutrition. 2019;59(2):196–206. doi: 10.1080/10408398.2017.1363710 28846446

[50] Yamashita T, Hashiramoto A, Haluzik M, Mizukami H, Beck S, Norton A, et al. Enhanced insulin sensitivity in mice lacking ganglioside GM3. Proceedings of the National Academy of Sciences. 2003;100(6):3445–9. doi: 10.1073/pnas.0635898100 12629211 PMC152312

[51] Inokuchi J-i, Kabayama K, Sato T, Igarashi Y. A New Pathological Feature of Insulin Resistance and Type 2 Diabetes: Involvement of Ganglioside GM3 and Membrane Microdomains. Sphingolipid Biology: Springer; 2006. p. 273–84.

[52] Sasaki N, Itakura Y, Toyoda M. Gangliosides contribute to vascular insulin resistance. International journal of molecular sciences. 2019;20(8):1819. doi: 10.3390/ijms20081819 31013778 PMC6515378

[53] Wishart DS, Feunang YD, Marcu A, Guo AC, Liang K, Vázquez-Fresno R, et al. HMDB 4.0: the human metabolome database for 2018. Nucleic acids research. 2018;46(D1):D608–D17. doi: 10.1093/nar/gkx1089 29140435 PMC5753273

[54] Satheesh G, Ramachandran S, Jaleel A. Metabolomics-Based Prospective Studies and Prediction of Type 2 Diabetes Mellitus Risks. Metabolic Syndrome and Related Disorders. 2020;18(1):1–9. doi: 10.1089/met.2019.0047 31634052

[55] Khoshnejat M, Banaei-Moghaddam AM, Kavousi K, Moosavi-Movahedi AA. Metabolic Modeling Reveals Potential Muscle Metabolite Markers of Insulin Resistance in Newly Diagnosed Diabetic Patients. 2020.

[56] Khoshnejat M, Banaei-Moghaddam AM, Kavousi K, Moosavi-Movahedi AA. In Silico Metabolic Modeling Reveals Potential Muscle Metabolite Markers of Insulin Resistance in Newly Diagnosed Diabetic Patients. 2020.

